# **Obituary for Ken Loi (7/7/69–28/10/23**)

**DOI:** 10.1007/s11695-024-07092-w

**Published:** 2024-03-06

**Authors:** Lilian Kow, Mark Magdy

**Affiliations:** 1 Adelaide, Australia; 2 Sydney, Australia

**Keywords:** Obituary, Ken Loi


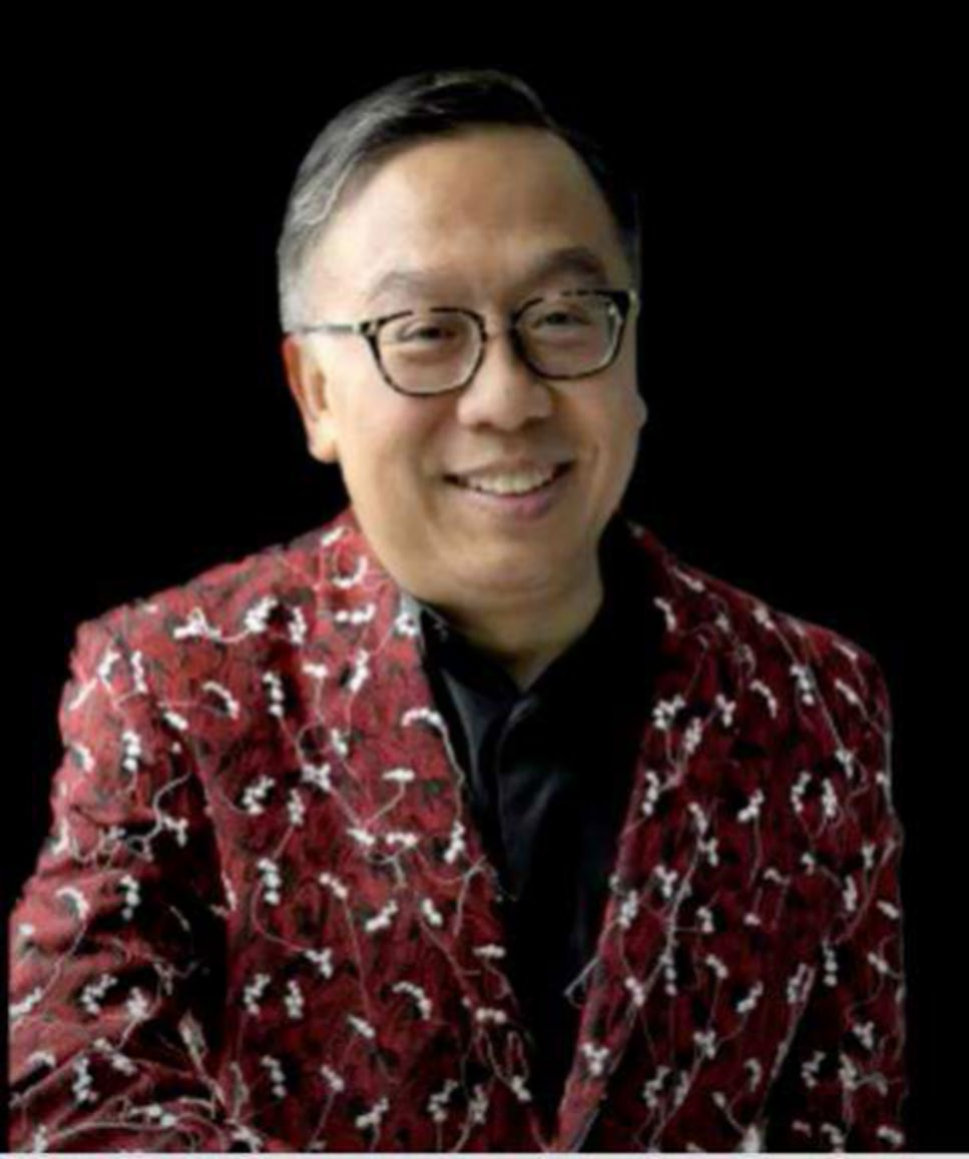


Dr Ken Loi was born in Hong Kong on 7 July 1969. He was an only child. He attended the famous and prestigious Wah Yan College, a Catholic all-boys institution run by the Society of Jesus in Wan Chai, Hong Kong.

He completed his high school education in Hong Kong and migrated, with his parents, to Sydney, Australia, in 1986. His medical journey began in 1988 where he studied medicine at the University of New South Wales. He graduated from medical school and completed an internship and residency in general surgery in Sydney. An exceptional trainee, Ken unfortunately lost his father during his surgical training. A testament to his organization and inner strength, he completed his general surgery training, cared for his family, and excelled in every aspect of his career.

Ken was a generational surgeon who was exceptional in his craft and was well-loved by all patients. He was also a deeply cherished member of the bariatric community both locally and worldwide. The strong foundation of The St George Obesity Program is testament to his unwavering commitment in the pursuit of excellence and the quality of care to his patients. In addition to his strong clinical ethos, Ken was an avid teacher who devoted his life to continuous education and mentorship.

Ken’s surgical practice spanned more than ten thousand procedures covering primary and revisional bariatric operations along with the management of benign and malignant upper gastrointestinal diseases. His expertise in bariatric surgery was instrumental in setting up the first Australian Centre of Excellence in St George Private Hospital. He was also accredited the status of Master Surgeon in bariatric surgery based on his extensive scope of bariatric cases.

Beyond his surgical practice, he was a regular speaker at both international and national conferences and has published widely in high-impact peer-review journals relating to bariatric and hernia surgery. Ken devoted his life to being a strong advocate for bariatric surgery and was heavily involved with both the national and international bariatric organizations. During his time as honorary treasurer of ANZMOSS, he was a key member in helping set up the Bariatric Surgical Registry in Australia and assisting the government in developing the public Medicare-funded bariatric service. In recognition of his tremendous service and contribution, Ken was awarded life membership of ANZMOSS in August 2023.

Being of an Asian background, Ken maintained a strong commitment and passion in developing bariatric surgery within the Asian Pacific region. He worked tirelessly within the IFSO-APC committee for more than a decade in helping set up and grow bariatric services within Asia. Prior to his passing, Ken was president-elect for IFSO-APC and was in line to become the next IFSO-APC President for 2023 until 2025.

Ken was also committed to the International Federation for the Surgery of Obesity and Metabolic Disorders (IFSO). He was very much part of the IFSO family with friends and colleagues in every continent. At the IFSO World Congress 2023 in Naples in August, he was awarded life membership to IFSO, an honor that was reflected by Ken’s unwavering dedication and commitment to the Federation and the discipline of metabolic and obesity medicine.

His other major achievements included being the first Asian Chair for RACS-NSW (2019–2021) and has been an Executive Member of RACS (Royal Australasian College of Surgeons) for more than 10 years. A substantial part of his role during his time within the college was devoted to advancing diversity within RACS and being part of the Surgical Sustainability Working Party in helping shape the next generation of surgeons. During COVID-19, he was also tasked with leading the college within New South Wales in responding to the management of the COVID crisis through the Community of Surgery Practice.

Beyond bariatric surgery, Ken also held a strong passion for hernia surgery and had played a key role in setting up ANZ Hernia Society. He had held the position of Executive Secretary and had been heavily involved with the education and publication of Advanced Minimally Invasive Hernia Surgery. This included hosting the regularly held hernia society journal club while playing an integral role in helping set up the Pilot Hernia Surgery Registry.

He was also a wonderful son to his mother who has survived losing her husband and now her only son, a doting father to Isabella, and a dedicated husband to Shirley who has shared much good times and of course the journey of his illness which he was so determined to outlive but could not do so.

Ken’s journey has touched many lives, near and afar.

His legacy will continue to live on through the memories shared and the many lives that he has touched—family, friends, colleagues, patients, and their families included.

We bid farewell to a remarkable surgeon and human being, Dr Ken Loi. In memory of his passing, we celebrate all the brilliant work that he has done for the surgical community within Australia and around the world. May all of his efforts be an inspiration to all surgeons for generations to come.

This is a dear friend and colleague that will be forever in our hearts, dearly missed but never forgotten.

May he now rest in peace.

Lilian Kow and Mark Magdy.

Australia.

